# A Model for Predicting Cation Selectivity and Permeability in AMPA and NMDA Receptors Based on Receptor Subunit Composition

**DOI:** 10.3389/fnsyn.2021.779759

**Published:** 2021-11-29

**Authors:** Sampath Kumar, Sanjay S. Kumar

**Affiliations:** ^1^College of Arts and Sciences, University of Pennsylvania, Philadelphia, PA, United States; ^2^Department of Biomedical Sciences, College of Medicine and Program in Neuroscience, Florida State University, Tallahassee, FL, United States

**Keywords:** ion selectivity, ion permeability, AMPA receptors, NMDA receptors, subunit composition, charge permeability equation

## Abstract

Glutamatergic AMPA (α-amino-3-hydroxy-5-methyl-4-isoxazolepropionic acid) and NMDA (N-methyl-D-aspartate) receptors are implicated in diverse functions ranging from synaptic plasticity to cell death. They are heterotetrameric proteins whose subunits are derived from multiple distinct gene families. The subunit composition of these receptors determines their permeability to monovalent and/or divalent cations, but it is not entirely clear how this selectivity arises in native and recombinantly-expressed receptor populations. By analyzing the sequence of amino acids lining the selectivity filters within the pore forming membrane helices (M2) of these subunits and by correlating subunit stoichiometry of these receptors with their ability to permeate Na^+^ and/or Ca^2+^, we propose here a mathematical model for predicting cation selectivity and permeability in these receptors. The model proposed is based on principles of charge attractivity and charge neutralization within the pore forming region of these receptors; it accurately predicts and reconciles experimental data across various platforms including Ca^2+^ permeability of GluA2-lacking AMPARs and ion selectivity within GluN3-containing di- and tri-heteromeric NMDARs. Additionally, the model provides insights into biophysical mechanisms regulating cation selectivity and permeability of these receptors and the role of various subunits in these processes.

## Highlights

- The general principles underlying cation selectivity and permeability in glutamatergic AMPA and NMDA receptors remain largely unknown.
- We present here a mathematical model that predicts the permeability of cations in these receptor channels based on their subunit composition.
- The model captures how subunit composition affects attractivity of ions and their electrostatic interactions with charged amino acids lining the channel pore which ultimately determine their permeability.
- Subunit-dependent cation selectivity represents a hitherto unrealized mechanism for finer control of Ca^2+^ influx enhancing the repertoire of synaptic AMPA and NMDA receptors.

## Introduction

Cation selectivity and permeability are important attributes of AMPA and NMDA receptor function impacting a variety of Na^+^/Ca^2+^-dependent cellular processes in the brain. While Ca^2+^ influx through NMDARs can bring about synaptic plasticity, the basis of learning and memory, or cell death from excitotoxicity (Collingridge, 1987; McBain and Mayer, 1994; Sheng et al., 1994; Cull-Candy et al., 2001), these receptors usually rely on Na^+^ influx through co-expressed AMPARs for the depolarization required to relieve them of their voltage dependent Mg^2+^ blockade for activation (Mayer et al., 1984). How do these receptors achieve this specificity for cations and what factors influence their permeability? The discovery of a heterogeneous population of receptor subtypes across different cell types and brain regions together with the assessment of their physiological properties under normal physiological conditions and in disease, all point to receptor subunit composition as a crucial determining factor in their selectivity for and permeability to cations. Note that while selectivity and permeability may be interdependent, they represent distinct aspects of ion influx. Thus, selectivity, referred to in this study as the ability of a receptor channel to screen extracellular ions (monovalent vs. divalent or cations vs. anions, for example), may be decoupled from permeability or the influx of ions from outside to inside of a cell through the channel, best exemplified by the NMDAR’s voltage dependent Mg^2+^ blockade. For these receptors at least, subunit dependent cation selectivity represents a departure from the commonly held belief that these glutamate-activated, voltage-dependent ionotropic channels are selective for both monovalent and divalent cations (Moriyoshi et al., 1991; Dingledine et al., 1999; Traynelis et al., 2010; Hansen et al., 2018; Beesley et al., 2020b). Lack of selectivity is, however, not the default state because ion channels are inherently selective for an ion species and single amino acid substitutions within their selectivity filters have been shown to drastically alter their ion-selective properties. For AMPARs on the other hand, posttranscriptional modification of the GluA2 subunit and a switch in their subunit composition to incorporate it early in development underlies their ability to exclude Ca^2+^ and become selective for Na^+^ (Burnashev et al., 1992; Kumar et al., 2002). This study sheds light on the organizing principles that govern subunit-dependent cation selectivity in these receptors.

Synaptic AMPA and NMDARs assemble as tetramers comprising of four subunits. All NMDARs contain one or more of the obligatory glycine-binding GluN1 subunits, which when assembled with glutamate-binding GluN2 (GluN2A-GluN2D) subunits of the same type (e.g., GluN1-2A-1-2A) give rise to diheteromeric NMDARs (*d*-NMDARs) that are selective for and permeable to both Na^+^ and Ca^2+^. When assembled with glycine-binding GluN3 (GluN3A or GluN3B) subunits (e.g., GluN1-3A-1-3A), *d*-NMDARs are no longer activatable by glutamate and are no longer permeable to Ca^2+^. Triheteromeric NMDARs, on the other hand, contain three different types of subunits (Sheng et al., 1994; Chazot and Stephenson, 1997; Luo et al., 1997; Kumar and Huguenard, 2003; Hatton and Paoletti, 2005; Tovar et al., 2013) and rely on the regulatory GluN3 subunit for their ion selective properties. Those receptors assembled without GluN3 (e.g., GluN1-2A-1-2B) are selective for and permeable to both Na^+^ and Ca^2+^, while those assembled with GluN3, designated as *t*-NMDARs (e.g., GluN1-2B-3A-2B), are selective for Ca^2+^ over Na^+^ for permeation and inward current (Pilli and Kumar, 2012, 2014; Kumar, 2016; Beesley et al., 2019). AMPARs like NMDARs are heteromultimeric comprising of glutamate receptor subunits 1–4 (GluA1–GluA4) with varying stoichiometries (Hollmann and Heinemann, 1994). Those lacking the GluA2 subunit have traditionally been associated with inhibitory interneurons (McBain and Dingledine, 1993; Jonas et al., 1994; Geiger et al., 1995; Zhou and Hablitz, 1998; Yin et al., 1999) and have been shown to be permeable to Ca^2+^ (Hollmann et al., 1991; Geiger et al., 1995; Jonas and Burnashev, 1995; Gu et al., 1996; Washburn et al., 1997) and expressed in excitatory neurons as well during early postnatal development (Kumar et al., 2002).

To understand the organizing principles of cation selectivity and permeability, we first analyzed the amino acid residues at homologous locations within the pore-forming M2 domains of proteins that constitute the selectivity filter in all members of the GluN1, GluN2, and GluN3 subunit families of the NMDA receptor. We then modeled the putative selectivity filters for NMDARs whose subunit composition could be correlated with experimental data regarding their Ca^2+^/Na^+^ permeabilities. This allowed us to conceptualize a *ring of partial negativity*, alluded to in the literature previously in the context of other receptor types (Imoto et al., 1988; Itier and Bertrand, 2001; Fritsch et al., 2011), that manifests from the alignment of polar amino acid residues and an appreciation of how this ring charge can be altered as a function of receptor subunit composition. Next, we examined how subunit-mediated changes in ring charge affected the attractivity of cations and what effect occupancy of the pore with various ions has on its diminution and neutralization. The model for cation selectivity and permeability discerned from these observations in NMDARs is validated by extending it to predict the behavior of AMPARs. Another overarching aim of this study is to address conflicting information in the literature regarding Ca^2+^ permeability of GluN3-containing NMDARs from the fresh perspective of ion selectivity (Perez-Otano et al., 2001; Chatterton et al., 2002; Matsuda et al., 2002; Pachernegg et al., 2012; Pilli and Kumar, 2012; Pankratov and Lalo, 2014; Otsu et al., 2019; Beesley et al., 2020b).

Cation permeability data for AMPA and NMDARs is obtained from the literature using a variety of methods ranging from imaging to electrophysiological and/or ion-substitution experiments using acute brain slices for native receptors and/or heterologous expression systems for recombinantly expressed receptors.

## Materials and Methods

### Sequence Data

The amino acid sequences near the M2 segments of NMDA and AMPA receptor subunits were obtained from the UniProtKB protein knowledgebase for Rattus norvegicus (RN, Rat) using accession numbers: P35439, Grin1 (GluN1); Q00959, Grin2a (GluN2A); Q00960, Grin2b (GluN2B); Q00961, Grin2c (GluN2C); Q62645, Grin2d (GluN2D); Q9R1M7, Grin3a (GluN3A); Q8VHN2, Grin3b (GluN3B); P19490, Gria1 (GluA1); P19491, Gria2 (GluA2); P19492, Gria3 (GluA3); P19493, Gria4 (GluA4). The sequences were aligned using the *Clustal Omega* program in UniProt.^1^

## Results

**FIGURE 1:**
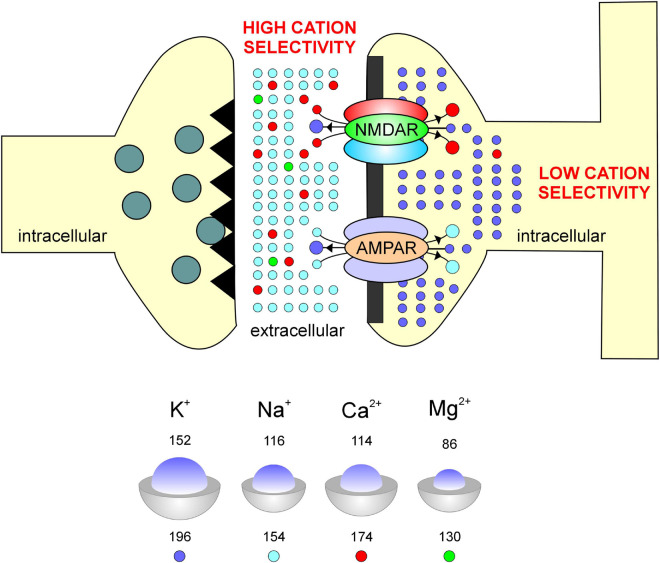
Schematic of a synapse compartmentalizing the intracellular and extracellular spaces as regions of differing cation selectivities for their permeation through AMPA and NMDARs. Factors affecting permeability and selectivity within these receptor channels include among others, the relative size of ions (inset blue, *top number*)/atoms (inset gray, *bottom number*), where numbers indicate radii in picometers.

Synaptic AMPA and NMDARs interface regions of high cation selectivity (the extracellular space referred to as synapse) with regions of low cation selectivity (the intracellular space within the spine head where the postsynaptic density is located) (Figure 1). At the synapse for example, the Na^+^ to Ca^2+^ concentration ratio is roughly a 10^2^ to 1 and yet, influx of ions through a calcium channel is mediated predominantly by Ca^2+^, requiring severe selection against Na^+^. In contrast, K^+^ to Ca^2+^ concentration within the intracellular compartment of a spine head is roughly a 10^6^ to 1 and so, discrimination against K^+^ is low (3,000:1) thereby affecting reversal potential which would be dominated by intracellular potassium rather than calcium concentration (Hille, 2001). Higher selectivity likely facilitates increased permeability on account of the electrostatic repulsion of a monovalent cation by a divalent cation and the speeding-up of divalent cation flow when the channel becomes multiply occupied (Sharma and Stevens, 1996). This taken together with the fact that unsolvated Na^+^ and Ca^2+^ ions are comparable in size (Shannon, 1976; *inset*, Figure 1), suggests that the increased permeability to Ca^2+^ and decreased permeability to Na^+^ in these receptor subtypes may arise from subunit-mediated cation selectivity.

### Selectivity Filters for NMDARs Vary With Subunit Composition

**FIGURE 2:**
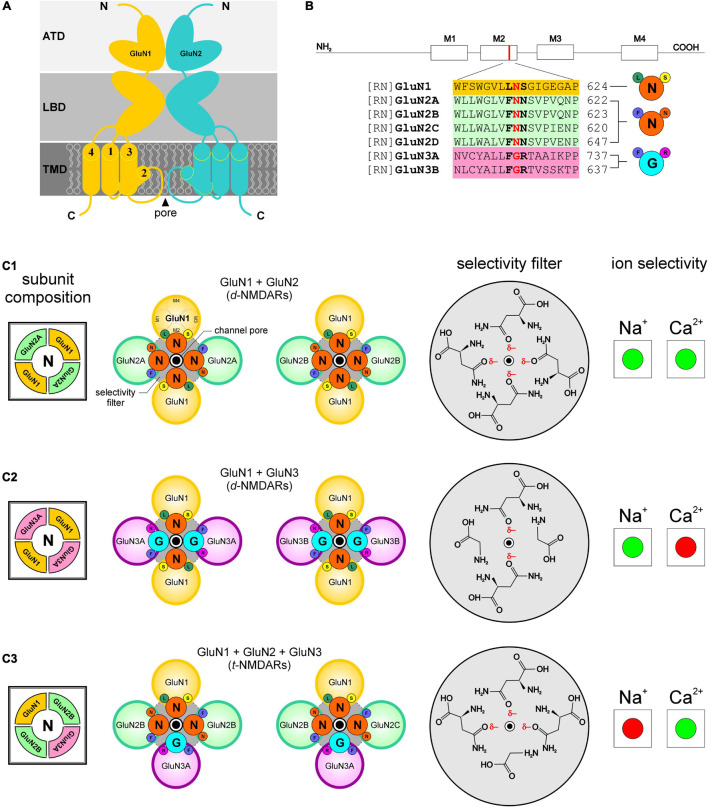
Selectivity for and permeability to cations in NMDARs is dependent on the combination of receptor subunits and ultimately on the sequence of amino acids that compose the selectivity filters in the pore-forming region of the receptor channels. Portions of this figure are taken from Beesley et al. (2019) to showcase differences in the selectivity filters of the identified receptors. **(A)** NMDAR schematic identifying various domains within subunits (ATD amino terminal domain, LBD ligand binding domain, TMD transmembrane domain; N, amino and C, carboxyl terminals) and the M2 helix which contributes to the pore-forming region of the receptor channel. **(B)** Predicted segments of the TMD and M2 sequence alignment of GluN1; GluN2A, 2B, 2C, 2D; GluN 3A, 3B from rat (RN, *rattus norvegicus*). Amino acid residues determining functional channel properties are indicated in bold and conserved residues determining ion selectivity in red. Numbers refer to amino acid positions within subunits. Abbreviations for the amino acid residues are: A, Ala; C, Cys; E, Glu; F, Phe; G, Gly; I, Ile; K, Lys; L, Leu; N, Asn; P, Pro; Q, Gln; R, Arg; S, Ser; T, Thr; V, Val; W, Trp; and Y, Tyr. **(C1–C3)** Schematic arrangement of subunits and amino acid residues that constitute the putative selectivity filters in the identified NMDARs (N, *left*), and their selectivity (enhanced, green; diminished, red) for Na^+^ and/or Ca^2+^ ions (*right*). Notice how the ring of partial negativity, arising from arrangement of partial negative charges (δ–) on asparagine’s polar side chains **(C1–C3)**, in the selectivity filter for GluN1 and GluN2-containing *d-*NMDARs (*gray circle*, top panel, **C_1_**; ring charge, ***r*_*q*_** = –4) is altered following changes in receptor subunit composition (middle panel, **C2**; ***r*_*q*_** = –2 and bottom panel, **C_3_**; ***r*_*q*_** = *–*3). The polar amino acids within the selectivity filters are depicted schematically such that they adopt energetically favorable orientations enabling their hydrophilic side chains to interact with the outside environment (i.e., the channel pore).

Site directed mutagenesis of amino acid residues within the pore-forming M2 domains of GluN1 and GluN2 subunits of heterologously expressed *d*-NMDARs (Figure 2A) shows that cation permeability, especially of divalent Ca^2+^, is mediated by asparagine at location 598 in GluN1 (N598) (Moriyoshi et al., 1991; Burnashev et al., 1992), the mandatory subunit (Monyer et al., 1994), and this residue is conserved at homologous positions within all members of the GluN2 subunit family (GluN2A-2D; Figure 2B). The coalescing of four partial negative charges (δ–), arising from asparagine’s more electronegative side-chain oxygen atom in each subunit of the tetrameric receptor, constitutes a *ring of partial negativity* that is conserved across all GluN2-containing di- and triheteromeric NMDARs. We acknowledge that asparagine’s partial negative charge may be distinct from the negative charge on an amino acid like glutamate or aspartate, however, we believe that these partial charges play an important role in electrostatic interactions underlying selectivity and are as such treated as full negative charges *in our model* (total ring charge *r*_*q*_ = –4). Together, these residues constitute the putative selectivity filter that enables permeation of both Na^+^ and Ca^2+^ ions through these receptor channels (Figure 2C1).

GluN3 subunits (3A and 3B), unlike GluN2, have non-polar glycine instead of asparagine at homologous positions within their M2 domains (Figure 2B) and expressing these subunits in the form of *d*-NMDARs disrupts the ring of partial negativity observed in GluN2-containing *d*-NMDARs and diminishes the total negative ring charge by 2 (*r*_*q*_ = –2; Figure 2C2). Expression of GluN1 and GluN3 subunits in *Xenopus* oocytes, HEK cells and juvenile hippocampal slices yields *d*-NMDARs that are activated by glycine instead of glutamate (Chatterton et al., 2002; Grand et al., 2018) and relatively Ca^2+^-impermeable (Chatterton et al., 2002; Matsuda et al., 2002) cf. (Otsu et al., 2019). Much of the current in these receptors is carried by Na^+^ instead of Ca^2+^ (Das et al., 1998; Matsuda et al., 2002; Figure 2C2).

Swapping a glycine-binding GluN1 subunit for a glycine-binding GluN3 subunit (3A or 3B) in GluN2-containing *d*-NMDARs yield *t*-NMDARs (Kumar, 2016) with a disrupted ring of partial negativity (*r*_*q*_ = –3; Figure 2C3). The incorporation of GluN3A in the subunit composition of diheteromeric GluN1/GluN2B-containing NMDARs causes a ∼5 to 10-fold reduction in NMDA-evoked Na^+^ current in oocytes (Sucher et al., 1995) which is consistent with what is also seen in *t*-NMDARs native to the brain (Pilli and Kumar, 2014; Beesley et al., 2020a,b). Together, these data suggest that without GluN3 control over selectivity of either monovalent (i.e., Na^+^) or divalent (i.e., Ca^2+^) cations (GluN3-containing *d*-NMDARs, however, appear to prefer Na^+^ over Ca^2+^) is diminished or lost and this situation is altered with the incorporation of GluN3 to make *t*-NMDARs, which acquire selectivity for Ca^2+^ over Na^+^ (Beesley et al., 2019, 2020b; Figure 2C3).

Dual regulation of monovalent and divalent cations in *t*-NMDARs is possible through ion selectivity (Premkumar and Auerbach, 1996; Schneggenburger, 1998) alone, or in combination with pore-size/conductance-based screening of ions (Burnashev et al., 1992; Wollmuth et al., 1996; Hille, 2001). Given the data from electrophysiological and/or ion-substitution experiments in the frontal, somatosensory and entorhinal cortices (Pilli and Kumar, 2012; Beesley et al., 2020b), together with the fact that unsolvated Na^+^ and Ca^2+^ ions are comparable in size (Shannon, 1976), suggests that the increased permeability to Ca^2+^ and decreased permeability to Na^+^ in *t*-NMDARs arises primarily from GluN3-mediated cation selectivity. We next examine how this selectivity for and permeability to cations might come about.

### Modeling the Permeability of Cations in *d*- and *t*-NMDARs

The permeability of a cation with charge **q_*i*_** (+1, monovalent; +2, divalent) through a *d*- or *t*-NMDAR channel is assumed to be determined by the yes/no piecewise function (1), where ***A***, defined as *charge attractivity* is multiplied by ***B***, defined as *inward drive*. Both ***A*** (2) and ***B*** (3) are themselves binary (0,1) piecewise functions of **q_*i*_**, ***r*_*q*_** (total ring charge of the selectivity filter) and/or **q_*r*_** (charge of ion occupying the pore).


(1) qipermeablity=A(rq,qi)×B(rq,qi,qr)={yes   if   1no     if   0

(2) $$\text{A}\left(r_{q},q_{i}\right)=\{\begin{array}{c} 1\;if\;\left|r_{q}+q_{i}\right|>0\\ 0\;if\;\left|r_{q}+q_{i}\right|=0 \end{array}$$

(3) $$\text{B}\left(r_{q},q_{i},q_{r}\right)=\{\begin{array}{c} 1\;if\;q_{i}+\left|r_{q}+q_{r}\right|>q_{r}\\ 0\;if\;q_{i}+\left|r_{q}+q_{r}\right|\leq q_{r} \end{array}$$

$$r_{q}=totalringchargeofselectivityfilter;\left(r_{q}\leq 0\right)$$

$$q_{i}=chargeofionenteringpore$$

$$q_{r}=chargeofionoccupyingpore$$

$$\text{A}\left(r_{q},q_{i}\right)=chargeattractivity$$

$$\text{B}\left(r_{q},q_{i},q_{r}\right)=inwarddrive$$

The *charge permeability Equation* (1) above is based on the premise that following receptor activation, **q_*i*_** should first be attracted to the selectivity filter based on the magnitude of its charge and the total ring charge of the selectivity filter ***r*_*q*_** (note that like charges repel and unlike charges attract). Once attractivity is established, **q_*i*_** experiences an inward drive depending on (1) charge of the ion occupying the pore, if any; (2) neutralization of ring charge in the selectivity filter on account of pore occupancy and (3) the net electromotive force exerted by **q_*i*_** on **q_*r*_** to displace it inwards. The model assumes that ion selectivity precedes permeability (increased selectivity is correlated with increased permeability) and associates a distinct, albeit unquantified, dwell time for ions within the pore of the receptor channels where they are temporarily trapped and held in place to enable electrostatic interactions with other ions. Note that this trap and hold scenario is distinct from the binding of ions to designated sites within the channel pore. The model does not consider concentration gradient, which would tend to move **q_*i*_** from the synapse into the cell through the receptor and affect the rate of permeation, affinity of ions for specific binding sites within the pore of the channel in such a manner as to block it (e.g., Mg^2+^) or membrane potential (V_*m*_, depolarized / hyperpolarized). It instead assumes that the receptor is fully activated following binding of the neurotransmitter and devoid of any Mg^2+^ blockade or desensitization such that cations have free access to its selectivity filter through the pore. Further modeling of receptor dynamics would account for (i) the nature of Mg^2+^ blockade and (ii) temporal changes in receptor channel conductance (Kumar and Huguenard, 2003). Finally, the output of the charge permeability equation is *binary* (1-permeable / 0-impermeable) and this information can be used as a prelude to the quantification of ion permeability across receptor channels (*graded*) using the Goldman-Hodgkin-Katz equations based on induced shifts in reversal potential (Mayer and Westbrook, 1987; Schneggenburger, 1996; Beesley et al., 2020b).

**FIGURE 3:**
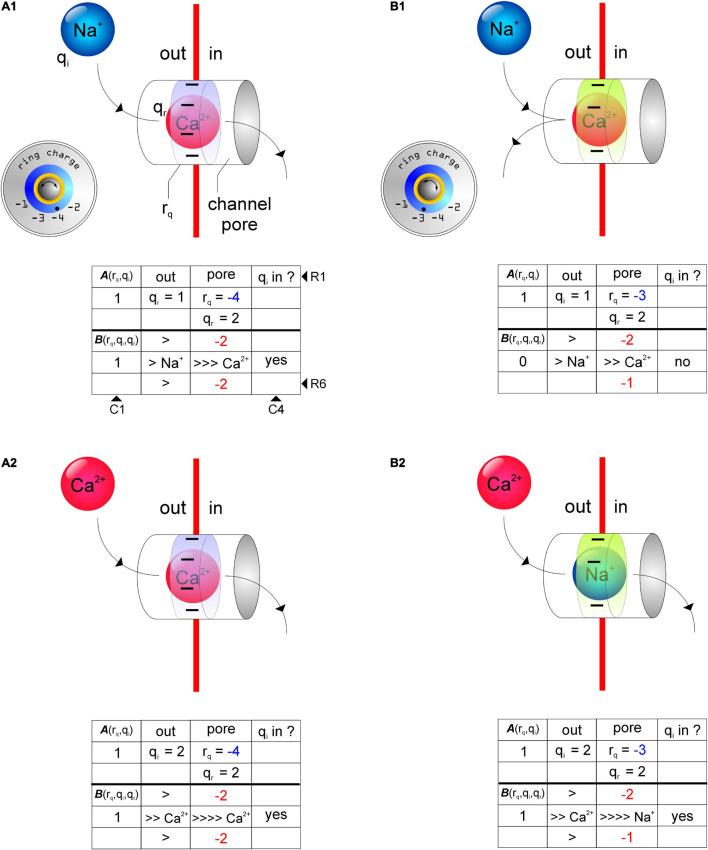
Schematics of test configurations depicting the use of the charge permeability equation (1) to predict selectivity and permeability of cations in receptor channels as a function of total ring charge of their respective selectivity filters (***r*_*q*_**, ring charge dials, *insets*). **(A_1_,A_2_)** Permeability of Na^+^ **(q_*i*_, A_1_)** and Ca^2+^ **(q_*i*_, A_2_)** in a receptor channel with ***r*_*q*_** = –4 when occupied by Ca^2+^ **(q_*r*_, A_1_, A_2_)**. **(B_1_, B_2_)** Permeability of Na^+^ (**q_*i*_**, **B_1_**) and Ca^2+^ (**q_*i*_**, **B_2_**) in a receptor channel with ***r*_*q*_** = –3 when occupied by Ca^2+^ (**q_*r*_, B_1_**) and Na^+^ (**q_*r*_, B_2_**). Note how Na^+^ can displace Ca^2+^ in a channel with ***r*_*q*_** = –4 but not ***r*_*q*_** = –3 **(A_1_,B_1_)** and that Ca^2+^ can displace itself or Na^+^ from the pore of a channel with ***r*_*q*_** = –4 or –3 **(A_2_,B_2_)**. The tables underneath the schematics depict the working underlying computation of the terms **A**(*r*_*q*_,*q*_*i*_)(R2C1; R, *row*; C, *column*) and **B**(*r*_*q*_,*q*_*i*_,*q*_*r*_) (R5C1) associated with the charge permeability equation. >, electromotive force on **q_*r*_** exerted by **q_*i*_** (R4-6C2, R5C3); numbers colored *red* (R4C3, R6C3), total ring charge **r_*q*_** (*blue*, R2C3) distributed around **q_*r*_** (R5C3).

**FIGURE 4:**
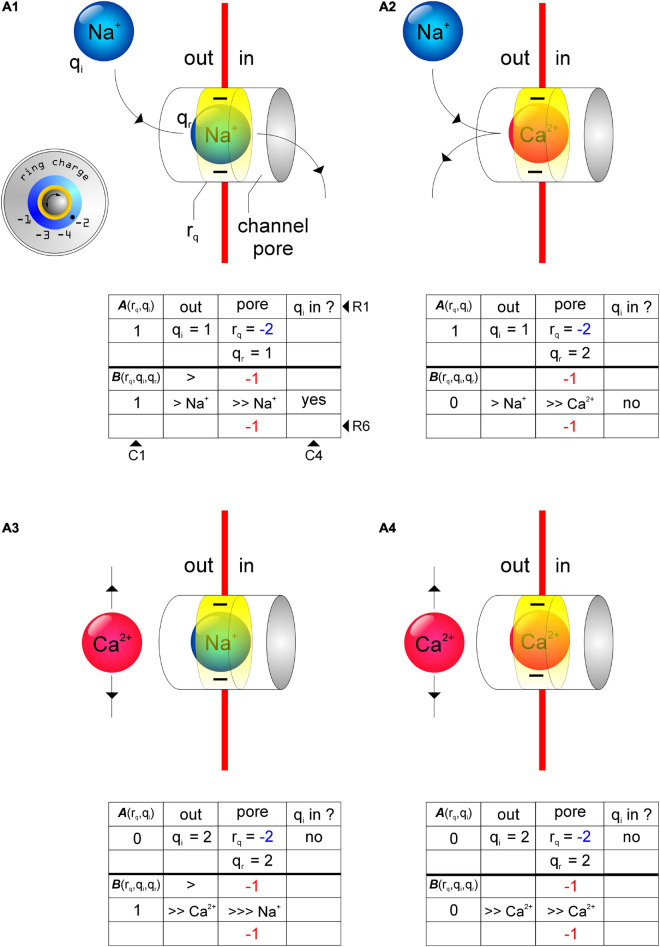
Schematics of test configurations depicting the use of the charge permeability equation (1) to predict selectivity and permeability of cations in receptor channels with ***r*_*q*_** = –2 (ring charge dial, *insets*). **(A_1_,A_2_)** Na^+^ (**q_*i*_**) can displace itself **(A_1_)**, but not Ca^2+^ **(A_2_)**, from the channel pore in receptors with ***r*_*q*_** = –2. **(A_3_,A_4_)** Attractivity of Ca^2+^ (**q_*i*_**) in these receptor channels is, however, diminished thereby rendering them Ca^2+^ impermeable. As before, tables underneath the schematics depict the working underlying computation of the terms **A**(*r*_*q*_,*q*_*i*_) (R2C1; R, *row*; C, *column*) and **B**(*r*_*q*_,*q*_*i*_,*q*_*r*_) (R5C1) associated with the charge permeability equation. >, electromotive force on **q_*r*_** exerted by **q_*i*_** (R4-6C2, R5C3); numbers colored *red* (R4C3, R6C3), total ring charge ***r*_*q*_** (*blue*, R2C3) distributed around **q_*r*_** (R5C3).

We assayed using the charge permeability Equation (1), the permeabilities of GluN2-containing *d*-NMDARs (***r*_*q*_** = –4; Figures 3A1,A2) and GluN3-containing *t*-NMDARs (***r*_*q*_** = –3; Figures 3B1,B2) to Na^+^ and Ca^2+^ under conditions when the pore is **(a)** empty, **(b)** occupied by Na^+^ and **(c)** occupied by Ca^2+^ (see Supplementary Figure 1). The schematics in Figure 3 depict various test configurations, the identities of **q_*i*_** and **q_*r*_** and the total ring charge of the respective selectivity filters, ***r*_*q*_** (indicated by *ring charge* dials in Figures 3A1,B1). The tables underneath the schematics depict the working underlying computation of ***A*** (R2C1; R, *row*; C, *column*) and ***B*** (R5C1) in determining **q_*i*_**’s permeability through the respective receptor channels [C4; >, electromotive force on **q_*r*_** exerted by **q_*i*_** (R4-6C2, R5C3); numbers colored *red* (R4C3, R6C3), total ring charge **r_*q*_** (*blue*, R2C3) distributed around **q_*r*_** (R5C3)]. The model predicts that both Na^+^ and Ca^2+^ can permeate GluN2-containing *d*-NMDARs and that these receptors are selective for both cations by virtue of their selectivity filter properties. This is a departure from the commonly held perspective that these receptors are non-selective for monovalent and divalent cations. Note that while both Na^+^ and Ca^2+^ can displace themselves or each other from within the pore of the *d*-NMDAR channel (Figures 3A1,A2), Na^+^ can displace Ca^2+^ only in *d*-NMDARs but not *t*-NMDARs due to the change in total ring charge ***r*_*q*_** (Figures 3A1,B1). Ca^2+^, on the other hand, can displace itself or Na^+^ in both *d*- and *t*-NMDARs (Figures 3A2,B2). This suggests that once the pore in a *t*-NMDAR becomes occupied by Ca^2+^, it becomes impermeable to Na^+^ and selective for Ca^2+^ (Figures 3B1,B2). A further reduction in **r_*q*_** from –3 to –2 (Figure 4) alters the ion selective properties of GluN3-containing *d*-NMDARs. For these receptors, the charge permeability equation (1) predicts selectivity for and permeability to Na^+^ when the pore is either empty or occupied by Na^+^ but not when occupied by Ca^2+^ (Figures 4A1,A2). Furthermore, selectivity for Ca^2+^ is diminished because of the loss of attractivity under all conditions of pore occupancy (***A*** = 0; Figures 4A3,A4), suggesting that these receptors are essentially selective for and permeable to Na^+^ but not Ca^2+^. Taken together, these data suggest that ion selectivity and permeability of NMDARs is dependent on the subunit-specific configuration of their selectivity filters and governed by the charge permeability Equation (1).

### Validation of the Charge Permeability Equation Using AMPARs

**FIGURE 5:**
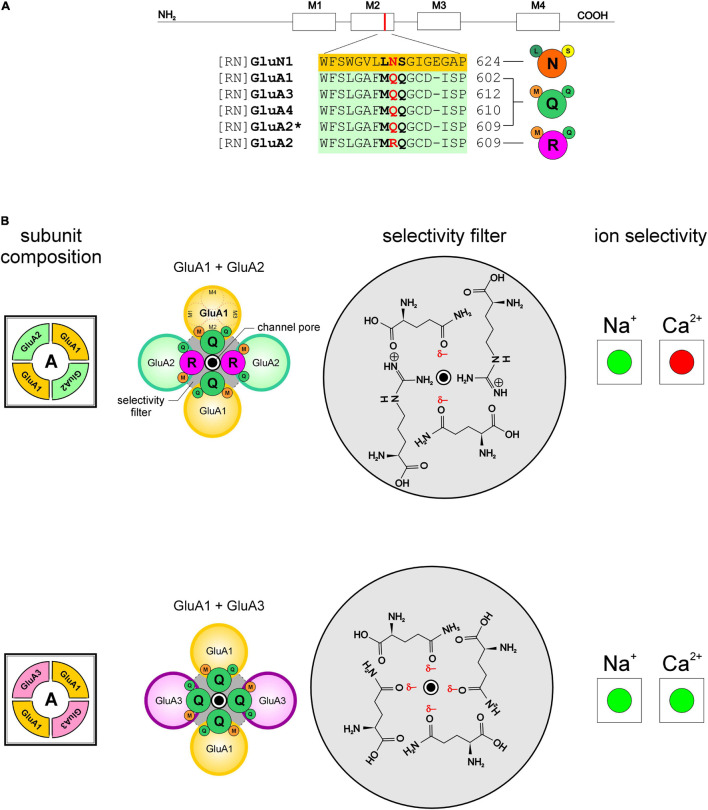
Selectivity and permeability of cations in AMPARs is dependent on the GluA2 subunit and the sequence of amino acids that compose the selectivity filters in the pore-forming region of the receptor channels. **(A)** Predicted membrane helices (M1-M4) and M2 sequence alignment of GluN1; GluA1, A3, A4, and A2*, post-transcriptionally modified form of A2 from rat (RN, *rattus norvegicus*). Amino acid residues determining functional channel properties are indicated in bold and conserved residues determining ion selectivity in red. Numbers refer to amino acid positions within subunits. Abbreviations for the amino acid residues are: A, Ala; C, Cys; E, Glu; F, Phe; G, Gly; I, Ile; K, Lys; L, Leu; N, Asn; P, Pro; Q, Gln; R, Arg; S, Ser; T, Thr; V, Val; W, Trp; and Y, Tyr. **(B)** Schematic arrangement of subunits and amino acid residues that constitute the putative selectivity filters in the identified AMPARs (A, *left*), and their selectivity (enhanced, green; diminished, red) for Na^+^ and/or Ca^2+^ ions (*right*). Notice how the ring of partial negativity, arising from arrangement of partial negative charges (δ–) on glutamine’s polar side chains, in the selectivity filter for GluA2-lacking AMPARs (*gray circle*, bottom panel; ring charge, ***r*_*q*_** = –4) is altered following its incorporation into the subunit composition of the receptor (*gray circle*, top panel ***r*_*q*_** = –2).

To validate the charge permeability equation, we tested its applicability in predicting Ca^2+^ permeability of AMPARs (Figure 5; for non-selective permeation of monovalent cations through AMPARs; see Biedermann et al., 2021). It is now well known that AMPARs assembled without the post-translationally modified GluA2 subunit are Ca^2+^ permeable unlike those assembled with it (Hollmann et al., 1991). The incorporation of GluA2 in the subunit composition of AMPARs introduces an arginine (R) in place of a glutamine (Q) at location 605 in the M2 domain of the subunit, thereby reducing the total ring charge of their selectivity filters, ***r*_*q*_** from –4 to –2 (Figures 5A,B). We know from NMDARs that a ***r*_*q*_** of –2 renders receptors selective for and permeable to Na^+^ but not Ca^2+^ (Figure 5B, *top panel*). However, AMPARs assembled from any subunits other than GluA2 yield receptors with selectivity filters that have a ***r*_*q*_** of –4 (Figure 5B, *bottom panel*) that are known to be selective for and permeable to both Na^+^ and Ca^2+^ (Hollmann et al., 1991; Kumar et al., 2002). Thus, the charge permeability equation formulated using NMDARs can be extended to elucidate the principles of cation selectivity and permeability in AMPARs.

### Cation Selectivity and Permeability in AMPA and NMDARs Is Regulated by the Ring Charge of Their Respective Selectivity Filters

**FIGURE 6:**
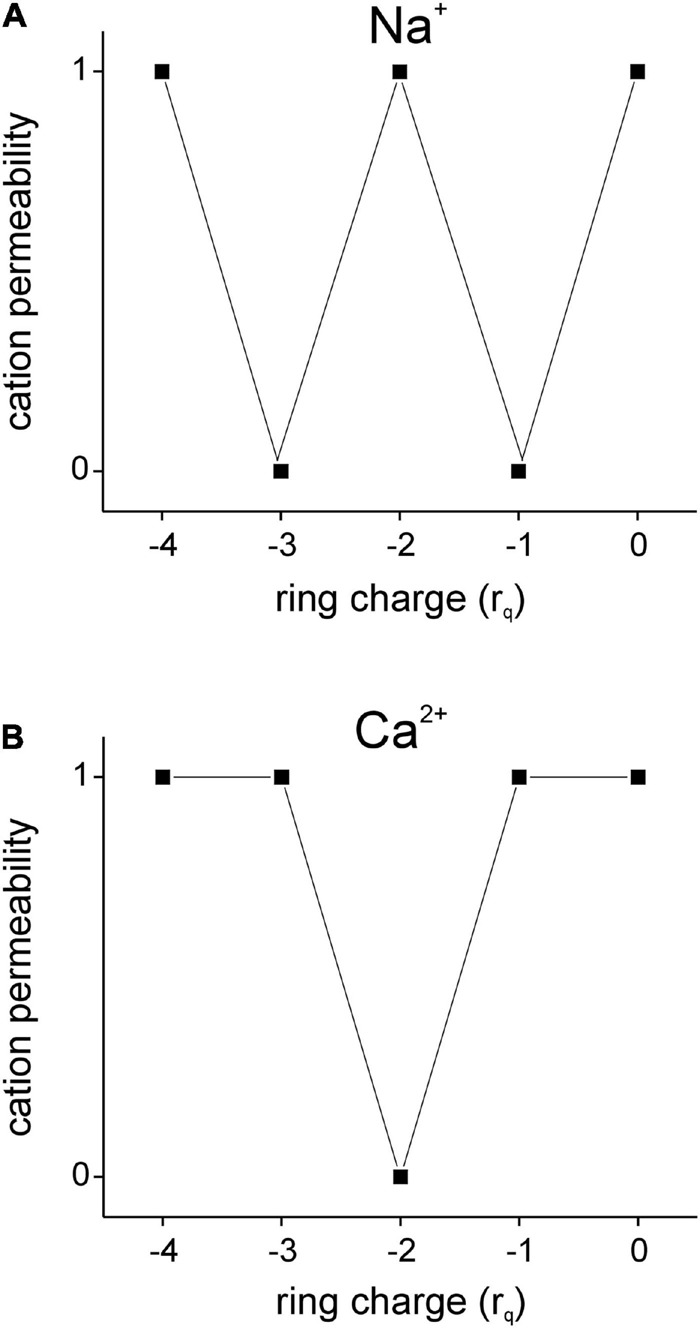
Permeability plots for monovalent (Na^+^, **A**) and divalent (Ca^2+^, **B**) cations as a function of total ring charge (***r*_*q*_**) are distinct from one another within the range –4 ≤ ***r*_*q*_** ≤ 0 as predicted by the charge permeability equation (1 = *permeable*; 0 = *not permeable*). Note that only when ***r*_*q*_** = –4 or 0 are AMPA and NMDA receptor channels fully permeable to both Na^+^ and Ca^2+^, but otherwise (–3 ≤ ***r*_*q*_** ≤ –1) are rendered selective for one cation over the other.

To fully characterize the permeabilities of Na^+^ and Ca^2+^ in AMPA and NMDARs and examine their dependence on receptor subunit composition, we plotted their permeabilities (1, *yes*; 0, *no*), as predicted by the charge permeability equation (1), as a function of total ring charge of their respective selectivity filters (Figure 6). Note that the permeability vs. ring-charge relationship for Na^+^ (Figure 6A) is distinct from that for Ca^2+^ (Figure 6B). For a tetrameric subunit configuration, permeability to both Na^+^ and Ca^2+^ is possible only when the selectivity filter has a ring of partial negativity that is either full (i.e., ***r*_*q*_** = –4) or empty (i.e., ***r*_*q*_** = 0). This may be the reason why *d*-NMDARs (***r*_*q*_** = –4) are selective for and permeable to Na^+^ and Ca^2+^. For all other combinations (–3 ≤ ***r*_*q*_** ≤ –1), the receptors are either permeable to Na^+^ or Ca^2+^, but not both. This selective permeability arises in part from the attractivity of the selectivity filters for various ions and the interactions ions occupying the pore of the channel have with its ring charge. For example, at ***r*_*q*_** = –3, Na^+^ is permeable through the receptor channel until it is displaced from the pore and occupied by Ca^2+^. Once occupied by Ca^2+^, the receptor becomes permeable only to Ca^2+^ because a calcium ion alone can displace it from the pore of the channel. This may be the reason why GluN3-containing *t*-NMDARs (***r*_*q*_** = –3) are highly Ca^2+^ permeable. At ***r*_*q*_** = –2, the attractivity to Ca^2+^ is zero and as Na^+^ can only displace Na^+^ but not Ca^2+^ from the pore of the channel, the receptor is rendered permeable only to Na^+^. This may be the reason why GluN2-containing AMPA and GluN3-containing *d*-NMDA receptors (***r*_*q*_** = –2) are permeable to Na^+^ but not Ca^2+^. At ***r*_*q*_** = –1, the attractivity to Na^+^ is reduced to zero, and as Ca^2+^ can displace either Na^+^ or Ca^2+^ from the pore of the channel, these receptors would be rendered permeable only to Ca^2+^. However, we know of no known AMPA or NMDARs with selectivity filters whose ***r*_*q*_** = –1.

## Discussion

This work describes a model for cation selectivity and permeability in AMPA and NMDA receptors based on receptor subunit composition. Cation selectivity is of importance given the diverse processes in which these receptors participate, especially those dependent on Ca^2+^. A developmental switch in subunit composition to incorporate a post-translationally modified GluA2 facilitates a switch in Ca^2+^ permeability of AMPARs on excitatory pyramidal neurons in the neocortex (Kumar et al., 2002), and failure to make this switch has been shown to predispose the cerebral cortex to hypoxic/ischemic injury in humans (Talos et al., 2006). GluN3 containing *t*-NMDARs in the entorhinal cortex have been implicated in Ca^2+^ induced excitotoxicity and loss of neurons in animal models of temporal lobe epilepsy, the most common form of epilepsy in adults, by virtue of their ability to select Ca^2+^ over Na^+^, making them more Ca^2+^ permeable than their non-GluN3 containing counterparts (Beesley et al., 2019, 2020b). Yet, GluN3-containing *d*-NMDARs have been shown to exhibit the opposite trend, preferring Na^+^ over Ca^2+^ for permeation (Sucher et al., 1995). From these and many other examples in the literature, it is becoming increasingly clear that cation selectivity is intimately related with receptor subunit composition and given that subunit stoichiometry, particularly for NMDARs, has been shown to change with age and across brain regions, cell types and synaptic inputs onto neurons (Kumar and Huguenard, 2003; Pilli and Kumar, 2012; Beesley et al., 2019), there is a need to better understand how subunit composition influences function and what the underlying principles are that link form to function.

### GluA2 and GluN3 Subunits Are Key Regulatory Elements of Cation Selectivity and Permeability in AMPA and NMDA Receptors, Respectively

This study explores the possibility that ion selectivity in these receptors arises from the attractivity of cations to a *ring of partial negativity* that manifests from the arrangement of partial negative charges on certain amino acids with polar uncharged hydrophilic side chains (asparagine, N; glutamine, Q; serine, S; threonine, T) that occupy fixed locations within the pore-forming M2 domains of each of the four subunits that compose these receptors. These amino acids are conserved across all members of a subunit family and their site directed mutagenesis has been shown to disrupt ion permeability in these receptors implying that they constitute a putative selectivity filter that enables them to screen for specific ions. Attractivity of cations by the selectivity filter ring charge, however, is not the sole determinant of their permeability. For example, Na^+^ is attracted to a receptor channel occupied by Ca^2+^ in both *d*-NMDARs (*r*_*q*_ = –4; Figure 3A1) and *t*-NMDARs (*r*_*q*_ = –3; Figure 3B1) but only in the *d-* but not *t*-NMDARs can Na^+^ displace Ca^2+^ from the channel pore. This may appear counterintuitive from the standpoint of Ca^2+^ being held in place more tightly in *d*- vs. *t*-NMDARs for Na^+^ to displace it from the pore on account of their larger ring charge, but the model predicts the opposite scenario. Thus, in addition to attractivity, the extent to which the ring charge is neutralized by the resident ion species, together with the magnitude of charges on the resident and entering ions collectively determine its permeability. By varying subunit stoichiometry AMPA and NMDARs effectively alter the ring charge of their selectivity filters and the dynamics of interaction with ambient cations that eventually leads to the emergence of ion selectivity and permeability. We believe the GluN3 subunit in NMDARs and the GluA2 subunit in AMPARs to be the key *regulatory elements* mediating changes in cation selectivity and permeability in these receptors. Indeed, subunit-dependent cation selectivity represents a hitherto unrealized mechanism for finer control of Ca^2+^ influx that further enhances the repertoire of synaptic AMPA and NMDARs.

### The Emergence of Cation Selectivity and Its Dependence on Receptor Subunit Composition

**FIGURE 7:**
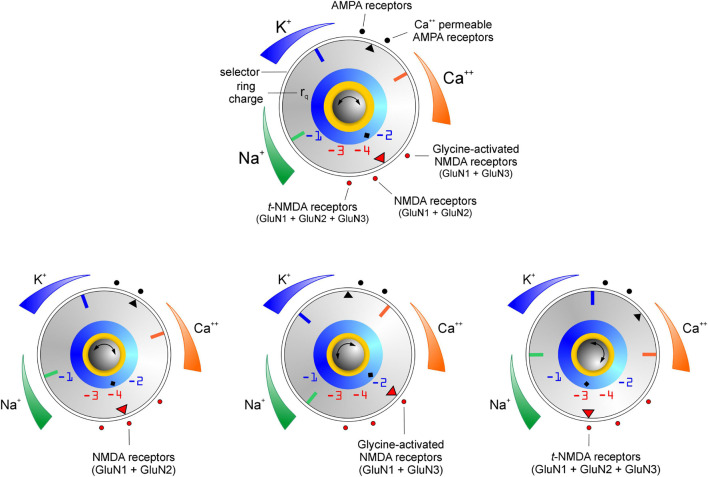
Schema for cation selectivity within ionotropic AMPA and NMDA receptors (*top panel*) with related examples (*bottom panels*). The rotary knob (center) is marked with a channel-selector (▼) and three orthogonally oriented indicators (blue, green and red) for read out of K^+^, Na^+^ and Ca^2+^ selectivity in the chosen receptor channels (•) arranged along the periphery of a selectivity dial (◆ indicates the ring charge ***r*_*q*_** associated with their selectivity filters). Cation selectivity levels which influence permeability, are *graded* (high ↔ low) and color-coded (non-colored regions indicate non-selective and/or impermeability). Note that emergence of Ca^2 +^ selectivity entails loss of Na^+^/K^+^ selectivity, however their permeability through these channels varies as a function of ***r*_*q*_** or receptor subunit composition.

We previously reported how evolutionarily conserved principles of cation selectivity in ion channels transcend the distinction of whether they are ligand-gated or not (Beesley et al., 2020b). The breakdown of a conserved signature sequence of amino acids (TVGYG) in the selectivity filters of highly K^+^-selective channels enables the emergence of Na^+^ and eventually, Ca^2+^ permeability in these channels which constitute a continuum of channel types with variable levels of K^+^, Na^+^, and Ca^2+^ selectivity (Zagotta, 2006). The empirically deduced schema for ion selectivity in ligand gated AMPA and NMDA receptor channels (Beesley et al., 2020b), hypothetically depicted with a “selectivity dial,” showing various receptor types (•; *red*, NMDA; *black*, AMPA) arranged along the outer periphery of a rotary dial marked with a receptor-selector (▼; *red*, NMDA; *black*, AMPA), and three orthogonally oriented indicators (**—**blue, green and red) for readout of K^+^, Na^+^ and Ca^2+^ selectivity now incorporates a ring charge indicator (◆, Figure 7 *top panel*) that enables correlation between the ring charge of the selectivity filter and permeability of the chosen receptors to Na^+^ and/or Ca^2+^. Cation selectivity levels are graded, color-coded (white/non-colored regions indicate non-selective and/or impermeant), and strategically positioned around the dial to capture the dynamics of change in ion selectivity as the knob is rotated to select for a particular receptor type and/or ring charge (Figure 7 *bottom panels*). Thus, it can be inferred that loss of K^+^ selectivity is associated with the emergence of Ca^2+^ permeability (Zagotta, 2006) and conversely increases in Ca^2+^selectivity come at the expense of corresponding decreases in Na^+^ permeability and alterations in ring charge of the selectivity filters.

Subunit-dependent ion selectivity and permeability, based on charge attractivity principles and negative electrostatic environment in the M2 region, has also been suggested for other ligand-gated ionotropic receptors including nicotinic acetylcholine receptors (nAChRs) and serotonin (5-HT_3_) receptors. However, the mechanisms proposed are all either structurally and/or functionally distinct from those indicated for AMPA and NMDARs in this study or are not fully understood. Neuronal nAChRs are pentameric structures whose subunits are classified into two families, alpha (α; α_2_–α_10_) and beta (β; β_2_–β_4_) (Itier and Bertrand, 2001). Homo-oligomeric α7 nAChRs primarily allow Ca^2+^ to flow into the cell when the channels open, whereas heteromeric (α_4_)_2_(β_2_)_3_ nAChR subtypes allow both Na^+^ and Ca^2+^ (Itier and Bertrand, 2001; Rang et al., 2003). The underlying reasons for this differential selectivity include, but are not restricted to, **(i)** the presence of a ring of negatively charged glutamate residues at the narrow intracellular end of the receptor (Imoto et al., 1988); **(ii)** differential effects of the hydrophobic constriction on water coordination by sodium and chloride ions; **(iii)** overall negative electrostatic environment in the M2 region and to some degree, the dynamics of the pore-lining residues and its crucial influence on the pore radius and ion hydration (Fritsch et al., 2011). Ion selectivity in the 5-HT_3_ receptors is under the control of charged rings at either end of the pore and dominated by the ring of negatively charged residues (glutamate) at the intracellular side of the channel. Changing the charge at this position has been shown to change ionic selectivity even in anion-selective receptors suggesting that electrostatic factors alone control selectivity in this family of receptors which also include nAChRs (Thompson and Lummis, 2003). Functional 5-HT_3_ channels may be homo (5-HT_3A_)- or heteropentameric (5-HT_(3A)1(3B–3E)4_) and are permeable to Na^+^, K^+^, and/or Ca^2+^ (Davies et al., 1999). To determine if the similarities in electrostatic mechanisms that control ion selectivity in the pentameric Cys-loop family of receptors (that include ACh and 5-HT_3_ receptors) also apply to AMPA and NMDARs, we increased the total negative ring charge of the selectivity filter ***r*_*q*_** to –5 and above (assuming receptors with hypothetical pentameric and hexameric subunit configurations), while querying for changes in ion selectivity and permeability using the charge permeability equation (1). Our model predicts that increasing the magnitude of the ring charge beyond –4 (***r*_*q*_** < –4) makes the receptors ineffective in screening for cations, enabling both Na^+^ and Ca^2+^ to permeate the channel. This built-in redundancy may be one reason why AMPA and NMDARs assemble as tetramers and not pentamers. Given that the ability to screen for ions is also lost when the ring charge is completely ablated (***r*_*q*_** = 0), suggests that cation selectivity and permeability in these receptors is also dominated by electrostatic mechanisms despite differences in the structural determinants of their selectivity filters.

### Can the Charge Permeability Equation Shed Light on the Permeation of Anions Through AMPA and NMDA Receptor Channels?

Finally, to test the predictive value of the proposed model (1), formulated primarily for assessing permeability of cations (Na^+^/Ca^2+^) in AMPA and NMDARs, we asked how subunit-driven alterations in ring charge of their selectivity filters affect their selectivity and permeability to anions (Cl^–^). The model predicts that full permeability to Cl^–^ alone does not arise until ***r*_*q*_** = –6 (see Supplementary Figure 2), suggesting that tetrameric configurations of AMPA and NMDARs with any of their known subunits are Cl^–^ impermeable. While attractivity is maintained throughout (***A*** = 1), the inward drive (***B***) varies depending on the ring charge and the ions occupying the pore such that in the presence of Na^+^ and Ca^2+^, AMPA and NMDARs are rendered impermeable to Cl^–^, which is consistent with what has been reported in the literature for *d*-NMDARs (Mayer and Westbrook, 1987; Sharma and Stevens, 1996). A Ca^2+^ occupying the pore of the channel occludes Cl^–^ for –5 ≤ ***r*_*q*_** ≤ 0 and a Na^+^ occupying the pore of the channel occludes Cl^–^ for –3 ≤ ***r*_*q*_** ≤ 0. Given that Na^+^ can displace a Cl^–^ from the channel pore under all conditions except ***r*_*q*_** = –1 and Ca^2+^ can displace a Cl^–^ from the channel pore under all conditions except ***r*_*q*_** = –2, the model predicts severe charge screening by anions for occupancy of the pore under these configurations. Thus, Na^+^ would have to compete with Cl^–^ for occupancy of the pore in AMPARs and GluN3 containing *d*-NMDARs. Furthermore, the model reconfirms the finding that homomeric AMPAR channels assembled from Q-form (***r*_*q*_** = –4) subunits are cation selective whereas those assembled from the R-form (***r*_*q*_** = 0) subunits are permeant to anions and cations suggesting that differences in the anion vs. cation selectivity, in Ca^2+^ permeability and in channel conductance are likely to be determined by the difference in charge density of the channel (Burnashev et al., 1996).

### Differences in the Behavior of Ca^2+^ and Mg^2+^ in NMDARs

Of note regarding selectivity and permeability of divalent cations through NMDARs are the differences in behavior of Ca^2+^ and Mg^2+^. Although the charge selectivity model does not discriminate between the two divalent cations, there needs to be an accounting of the differences in the directionality of their movement, the fact that Mg^2+^ is sensitive to changes in membrane voltage but Ca^2+^ is not, and whether the pore occupancy of one affects the selectivity of the other. It is generally believed that the binding site for Mg^2+^ is distinct from that for Ca^2+^ and located deep within the electrostatic field of the pore thereby exhibiting a marked voltage dependence of binding in contrast with the more superficial binding location for Ca^2+^ (Sharma and Stevens, 1996). Although the exact binding site for Ca^2+^ is not known, this study highlights two other predictive aspects on which the model (1) is based, namely subunit-specific attractivity of ions and charge interactions within the pore of the receptor channels. Substitution of asparagine with glutamine at location 614 (N614Q) in the ε1 subunit has no effect on Ca^2+^ permeability but strongly reduces the affinity of Mg^2+^ for its binding site due to an increase in the rate of unbinding. The same mutation in the ζ1 subunit, on the other hand, reduces both the permeability of the channel for Ca^2+^ ions and its block by Mg^2+^ (Moriyoshi et al., 1991; Meguro et al., 1992; Monyer et al., 1992; Yamazaki et al., 1992). The presence of Ca^2+^ within the channel pores block the flux of sodium and other ions highlighting the emergence of ion selectivity and as a single mutation perturbs two distinct binding sites for Mg^2+^ and Ca^2+^ in opposite ways, the binding of divalent ions at the two sites interact with one another (Sharma and Stevens, 1996). In summary, the model proposed provides mechanistic insights into the processes that govern ion selectivity and permeability in AMPA and NMDARs and to some extent reconciles data pertaining to them across various experimental platforms.

## Data Availability Statement

The original contributions presented in the study are included in the article/Supplementary Material, further inquiries can be directed to the corresponding author/s.

## Author Contributions

SK helped in conceptualization, data analysis, and mathematical modeling. SSK helped in conceptualization, mathematical modeling, and writing of the manuscript. Both authors contributed to the article and approved the submitted version.

## Conflict of Interest

The authors declare that the research was conducted in the absence of any commercial or financial relationships that could be construed as a potential conflict of interest.

## Publisher’s Note

All claims expressed in this article are solely those of the authors and do not necessarily represent those of their affiliated organizations, or those of the publisher, the editors and the reviewers. Any product that may be evaluated in this article, or claim that may be made by its manufacturer, is not guaranteed or endorsed by the publisher.

## Footnotes

1
https://www.uniprot.org/align
