# Supplementary material for: A Model for Predicting Cation Selectivity and Permeability in AMPA and NMDA Receptors Based on Receptor Subunit Composition
Source: Front Synaptic Neurosci. 2021 Nov 29;13:779759. doi: 10.3389/fnsyn.2021.779759 (PMC8667807; doi:10.3389/fnsyn.2021.779759)
Supplement: Supplementary file 1 [file Data_Sheet_1.docx]

ion occupying pore

ion permeability

inward drive

total ring charge of

selectivity filter

ion entering pore

charge of ion entering pore

charge of ion occupying pore

charge attractivity

|  |  |  |  |  |  |  |  |
| --- | --- | --- | --- | --- | --- | --- | --- |
| **q_i_** | **q_r_** | **r_q_** | **q_i_** | **q_r_** | **A** | **B** | **AB** |
|  |  |  |  |  |  |  |  |
| Na^+^ |  | -4 | 1 | 0 | 1 | 1 | YES |
| Na^+^ | Na^+^ | -4 | 1 | 1 | 1 | 1 | YES |
| Na^+^ | Ca^2+^ | -4 | 1 | 2 | 1 | 1 | YES |
| Ca^2+^ |  | -4 | 2 | 0 | 1 | 1 | YES |
| Ca^2+^ | Na^+^ | -4 | 2 | 1 | 1 | 1 | YES |
| Ca^2+^ | Ca^2+^ | -4 | 2 | 2 | 1 | 1 | YES |
|  |  |  |  |  |  |  |  |
| Na^+^ |  | -3 | 1 | 0 | 1 | 1 | YES |
| Na^+^ | Na^+^ | -3 | 1 | 1 | 1 | 1 | YES |
| Na^+^ | Ca^2+^ | -3 | 1 | 2 | 1 | 0 | NO |
| Ca^2+^ |  | -3 | 2 | 0 | 1 | 1 | YES |
| Ca^2+^ | Na^+^ | -3 | 2 | 1 | 1 | 1 | YES |
| Ca^2+^ | Ca^2+^ | -3 | 2 | 2 | 1 | 1 | YES |
|  |  |  |  |  |  |  |  |
| Na^+^ |  | -2 | 1 | 0 | 1 | 1 | YES |
| Na^+^ | Na^+^ | -2 | 1 | 1 | 1 | 1 | YES |
| Na^+^ | Ca^2+^ | -2 | 1 | 2 | 1 | 0 | NO |
| Ca^2+^ |  | -2 | 2 | 0 | 0 | 1 | NO |
| Ca^2+^ | Na^+^ | -2 | 2 | 1 | 0 | 1 | NO |
| Ca^2+^ | Ca^2+^ | -2 | 2 | 2 | 0 | 0 | NO |
|  |  |  |  |  |  |  |  |
| Na^+^ |  | -1 | 1 | 0 | 0 | 1 | NO |
| Na^+^ | Na^+^ | -1 | 1 | 1 | 0 | 0 | NO |
| Na^+^ | Ca^2+^ | -1 | 1 | 2 | 0 | 0 | NO |
| Ca^2+^ |  | -1 | 2 | 0 | 1 | 1 | YES |
| Ca^2+^ | Na^+^ | -1 | 2 | 1 | 1 | 1 | YES |
| Ca^2+^ | Ca^2+^ | -1 | 2 | 2 | 1 | 1 | YES |
|  |  |  |  |  |  |  |  |
| Na^+^ |  | 0 | 1 | 0 | 1 | 1 | YES |
| Na^+^ | Na^+^ | 0 | 1 | 1 | 1 | 1 | YES |
| Na^+^ | Ca^2+^ | 0 | 1 | 2 | 1 | 1 | YES |
| Ca^2+^ |  | 0 | 2 | 0 | 1 | 1 | YES |
| Ca^2+^ | Na^+^ | 0 | 2 | 1 | 1 | 1 | YES |
| Ca^2+^ | Ca^2+^ | 0 | 2 | 2 | 1 | 1 | YES |
|  |  |  |  |  |  |  |  |
| Na^+^ |  | -5 | 1 | 0 | 1 | 1 | YES |
| Na^+^ | Na^+^ | -5 | 1 | 1 | 1 | 1 | YES |
| Na^+^ | Ca^2+^ | -5 | 1 | 2 | 1 | 1 | YES |
| Ca^2+^ |  | -5 | 2 | 0 | 1 | 1 | YES |
| Ca^2+^ | Na^+^ | -5 | 2 | 1 | 1 | 1 | YES |
| Ca^2+^ | Ca^2+^ | -5 | 2 | 2 | 1 | 1 | YES |

**Supplementary Figure 1**. Predictions of the charge permeability equation (1) for the selectivity and permeability of Na^+^ and Ca^2+^ in AMPA and NMDA receptor channels under various ionic configurations as a function of total ring charge **r_q_**.

charge attractivity

charge of ion occupying pore

charge of ion entering pore

ion entering pore

total ring charge of

selectivity filter

inward drive

ion permeability

ion occupying pore

| **q_i_** | **q_r_** | **r_q_** | **q_i_** | **q_r_** | **A** | **B** | **AB** |
| --- | --- | --- | --- | --- | --- | --- | --- |
|  |  |  |  |  |  |  |  |
| Cl^-^ |  | -4 | -1 | 0 | 1 | 1 | YES |
| Cl^-^ | Na^+^ | -4 | -1 | 1 | 1 | 1 | YES |
| Cl^-^ | Ca^2+^ | -4 | -1 | 2 | 1 | 0 | NO |
| Cl^-^ | Cl^-^ | -4 | -1 | -1 | 1 | 1 | YES |
|  |  |  |  |  |  |  |  |
| Cl^-^ |  | -3 | -1 | 0 | 1 | 1 | YES |
| Cl^-^ | Na^+^ | -3 | -1 | 1 | 1 | 0 | NO |
| Cl^-^ | Ca^2+^ | -3 | -1 | 2 | 1 | 0 | NO |
| Cl^-^ | Cl^-^ | -3 | -1 | -1 | 1 | 1 | YES |
|  |  |  |  |  |  |  |  |
| Cl^-^ |  | -2 | -1 | 0 | 1 | 1 | YES |
| Cl^-^ | Na^+^ | -2 | -1 | 1 | 1 | 0 | NO |
| Cl^-^ | Ca^2+^ | -2 | -1 | 2 | 1 | 0 | NO |
| Cl^-^ | Cl^-^ | -2 | -1 | -1 | 1 | 1 | YES |
|  |  |  |  |  |  |  |  |
| Cl^-^ |  | -1 | -1 | 0 | 1 | 0 | NO |
| Cl^-^ | Na^+^ | -1 | -1 | 1 | 1 | 0 | NO |
| Cl^-^ | Ca^2+^ | -1 | -1 | 2 | 1 | 0 | NO |
| Cl^-^ | Cl^-^ | -1 | -1 | -1 | 1 | 1 | YES |
|  |  |  |  |  |  |  |  |
| Cl^-^ |  | 0 | -1 | 0 | 1 | 0 | NO |
| Cl^-^ | Na^+^ | 0 | -1 | 1 | 1 | 0 | NO |
| Cl^-^ | Ca^2+^ | 0 | -1 | 2 | 1 | 0 | NO |
| Cl^-^ | Cl^-^ | 0 | -1 | -1 | 1 | 1 | YES |
|  |  |  |  |  |  |  |  |
| Cl^-^ |  | -5 | -1 | 0 | 1 | 1 | YES |
| Cl^-^ | Na^+^ | -5 | -1 | 1 | 1 | 1 | YES |
| Cl^-^ | Ca^2+^ | -5 | -1 | 2 | 1 | 0 | NO |
| Cl^-^ | Cl^-^ | -5 | -1 | -1 | 1 | 1 | YES |
|  |  |  |  |  |  |  |  |
| Cl^-^ |  | -6 | -1 | 0 | 1 | 1 | YES |
| Cl^-^ | Na^+^ | -6 | -1 | 1 | 1 | 1 | YES |
| Cl^-^ | Ca^2+^ | -6 | -1 | 2 | 1 | 1 | YES |
| Cl^-^ | Cl^-^ | -6 | -1 | -1 | 1 | 1 | YES |
|  |  |  |  |  |  |  |  |
| Cl^-^ |  | -7 | -1 | 0 | 1 | 1 | YES |
| Cl^-^ | Na^+^ | -7 | -1 | 1 | 1 | 1 | YES |
| Cl^-^ | Ca^2+^ | -7 | -1 | 2 | 1 | 1 | YES |
| Cl^-^ | Cl^-^ | -7 | -1 | -1 | 1 | 1 | YES |

**Supplementary Figure 2**. Predictions of the charge permeability equation (1) for the selectivity and permeability of Cl^-^ in AMPA and NMDA receptor channels under various ionic configurations as a function of total ring charge **r_q_**.
